# Glycine increases preimplantation development of mouse oocytes following vitrification at the germinal vesicle stage

**DOI:** 10.1038/srep37262

**Published:** 2016-11-15

**Authors:** Xin-Yan Cao, Jack Rose, Shi-Yong Wang, Yong Liu, Meng Zhao, Ming-Jie Xing, Tong Chang, Baozeng Xu

**Affiliations:** 1 Institute of Special Animal and Plant Sciences, Chinese Academy of Agricultural Sciences, No.4899 Juye Street, Jingyue District, Changchun 130112, China; 2 State Key Laboratory for Molecular Biology of Special Economic Animal and Plant Science, Chinese Academy of Agricultural Sciences, Changchun 130112, P. R. China; 3 Idaho State University, Department of Biological Sciences, Pocatello, 83209, USA; 4 Key Laboratory of Embryo Development and Reproductive Regulation of Anhui Province, College of Biological and Food Engineering, Fuyang Teachers College, Fuyang, China

## Abstract

Ice-free cryopreservation, referred to as vitrification, is receiving increased attention in the human and animal assisted reproduction. However, it introduces the detrimental osmotic stress by adding and removing high contents of cryoprotectants. In this study, we evaluated the effects of normalizing cell volume regulation by adding glycine, an organic osmolyte, during vitrification of mouse germinal vesicle stage oocyte and/or subsequent maturation on its development. The data showed that glycine supplementation in either vitrification/thawing or maturation medium significantly improved the cytoplasmic maturation of MII oocytes manifested by spindle assembly, chromosomal alignment, mitochondrial distribution, euploidy rate, and blastocyst development following fertilization *in vitro*, compared to the control without glycine treatment. Furthermore, glycine addition during both vitrification/thawing and maturation further enhanced the oocyte quality demonstrated by various markers, including ATP contents and embryo development. Lastly, the effect of anti-apoptosis was also observed when glycine was added during vitrification. Our result suggests that reducing osmotic stress induced by vitrification could improve the development of vitrified mouse oocyte.

Cryopreservation of oocytes, is standard practice in Assisted Reproductive Technologies^1^,^2^,^3^ (ART). Indeed, the experimental connotation, once associated with oocyte cryopreservation has been removed^4^,^5^. Pregnancy rates with cryopreserved oocytes equal those using fresh or cryopreserved embryos and the incidence of birth anomalies does not differ between them^6^,^7^,^8^. The use of oocytes avoids ethical concerns and legal restrictions associated with embryo preservation. It reduces the risk and costs of repeated ovarian stimulation, since excess oocytes can be stored. Freezing oocytes is attractive to women without a partner or soon to lose ovarian function due to surgery, chemotherapy or radiation. In addition, women are increasingly choosing to delay motherhood because of demanding careers and/or financial concerns. In support, some companies (*ie*. Apple and Facebook) have offered to sponsor women employees who choose to freeze their eggs to delay pregnancy^9^.

Historically, oocytes were frozen in the second metaphase (MII) of meiosis (mature oocytes). Unfortunately, MII oocytes are very sensitive to the destructive effects of freezing because of their high membrane permeability and complicated constructions. Freezing induced damages include loss of cell membrane, meiotic spindle disorganization, chromatid disjunction and aneuploidy^10^,^11^,^12^, abnormal mitochondrial distribution and activity (*ie.* ATP production), premature cortical granule release and zona pellucida hardening^3^,^13^,^14^,^15^.

To circumvent the problems associated with MII oocytes, research efforts have recently focused on the cryopreservation of germinal vesicle (GV) stage, or immature oocytes^14^. Because GV oocytes have not yet developed a spindle apparatus, they are thought to be more resistant to freezing-induced damage. Nevertheless, GV oocytes are susceptible to freezing inflicted injury and their rate of development into blastocysts remains low compared to MII oocytes^16^. A major difficulty in the use of frozen GV oocytes is that after thawing, they must undergo *in vitro* maturation in order to be fertilized and develop into embryos^17^,^18^. The optimal incubation media that will ensure successful *in vitro* maturation of GV oocytes (developmental competence), has yet to be established.

Oocyte freezing is often accomplished through vitrification, an ice-free cryopreservation process achieved by ultra-rapid cooling rates in a small volume of media^19^. Unfortunately, the concentrations of cryoprotectants required for vitrification are much greater than those used in slow cooling, resulting in a hypertonic environment and abnormal osmotic pressures that are detrimental to cell viability^20^,^21^.

Physiologically, embryos appear to maintain cell volume as they pass through the oviducts and uterus by first importing inorganic ions and later small organic molecules called osmolytes^22^. Some putative organic osmolytes, are amino acids or amino acid derivatives such as glutamine, glycine, betaine, proline and beta-alanine. Of these, glycine appears to convey the greatest level of protection against the hypertonicity created by the cryoprotectants^23^,^24^. Moreover, a single powerful transporter (GLYT1) for glycine is present and active in embryos until compaction^25^. Using MII oocytes, or 1 and 2-cell stage mouse embryos, it was shown that the accumulation of glycine varied proportionately with the osmolarity of the incubation media^26^,^27^,^28^. Moreover, glycine supplementation, allowed 1-cell stage mouse embryos to develop through the 2-cell stage block in media that was 70 mOsM more hypertonic when compared to media without glycine^24^,^27^. Inhibition of GLYT1 completely abolishes the development of 1-cell mouse embryos in hypertonic media as well as osmosensitive glycine accumulation^27^. Glycine supplementation of mouse MII oocytes during vitrification maintained mitochondrial distribution, mitochondrial membrane potential and development to the blastocyst stage, when compared to those without glycine^29^. Collectively, these findings suggest that embryonic cells take up glycine, in part, to balance external osmolarity.

While GLYT1 activity is undetectable in GV oocytes *in situ* (which also contain very little endogenous glycine), transporter activity is induced upon ovulation or soon after removal from the ovarian follicle^28^. It would not be unreasonable to propose that glycine supplementation during *in vitro* maturation, might increase the developmental competence of the gametes. Therefore our objectives were to determine the effects of glycine supplementation during vitrification and *in vitro* maturation on the developmental competance of GV oocytes, as determined by (1). Oocyte survivability and progression to the blastocyst stage, (2). Assembly of meiotic spindle and chromosomal allignment, (3). Rate of oocyte aneuploidy, (4). Mitochondrial distribution and activity (ATP production), and (5). Rate of apoptosis.

## Results

### Glycine supplementation improved the preimplantation development following COCs vitrification

**Figure 1:**
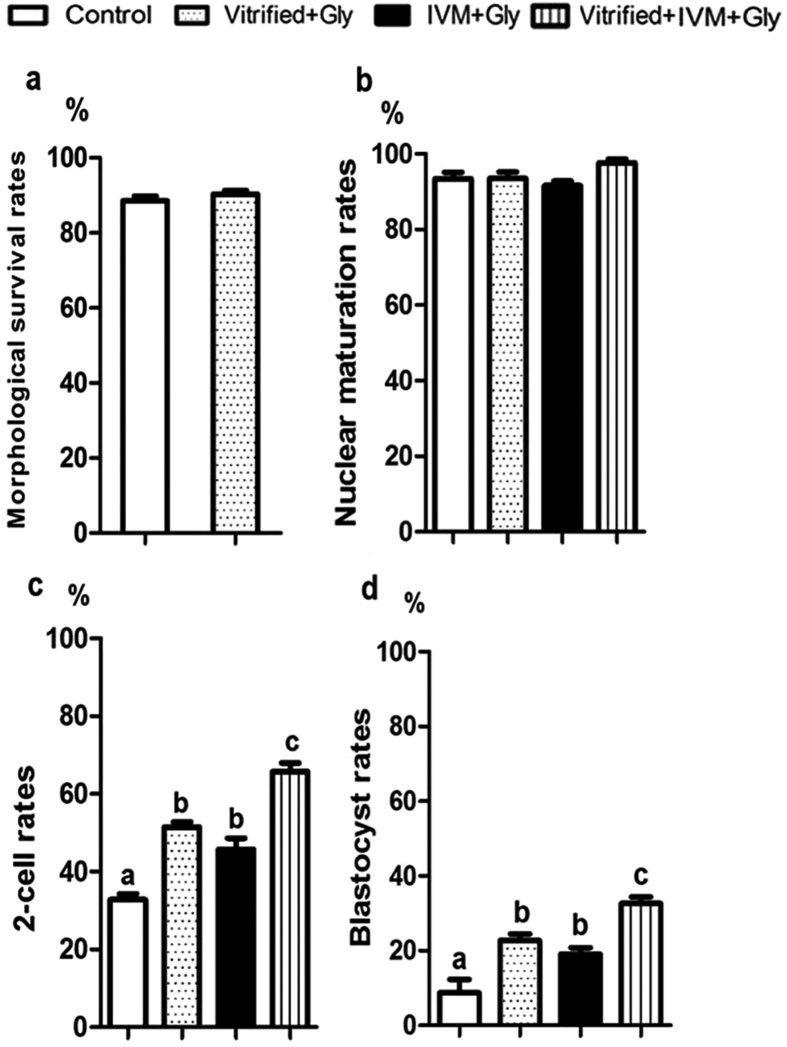
Effects of Glycine supplementation during vitrification and/or *in vitro* maturation on oocytes’ survival, maturation and subsequent preimplantation development. (**a**) The survival rate of vitrified GV oocyte with or without glycine addition during vitrification/thawing. (**b**) The percentage of MII oocytes (number of MII oocytes/number of survival GV oocytes) from various glycine treatment groups. (**c**) The rate of 2-cell embryo (number of 2-cell embryos/number of MII oocytes) after *in vitro* fertilization. d. The percentage of blastocyst (number of blastocysts/number of 2-cell embryos) from different glycine treatment groups. Control: without glycine treatment; IVM + Gly: glycine treatment during *in vitro* maturation only; Vitrified + Gly: glycine treatment during vitrification/thawing only; Vitrified + IVM + Gly: glycine treatment during both vitrification/thawing and maturation *in vitro*. Different low case letters above columns indicate statistical differences at *P* < 0.05.

To test whether glycine supplementation in the vitrification and thawing medium increases the survival rate of oocytes at GV stage, we freezed and thawed the COCs with or without physiological levels of glycine at 1 mM, which was found to be effective in maintaining normal cell volume in previous study, respectively. The results indicated that there was no significant difference in the survival rate (*P* > 0.05, N = 5) of oocytes, manifested by whether they had evenly granulated cytoplasm with apparent perivitelline space (viable) or degenerated cytoplasm without perivitelline space (nonviable), between glycine (90.3 ± 1.0%, n = 141) and control (88.5 ± 1.3%, n = 141) groups (Fig. 1a). To further examined whether glycine supplementation improves the oocyte maturation following COCs vitrification, we compared the nuclear maturation rate among groups of adding 1 mM glycine into either vitrification medium or maturation medium, or both vitrification and maturation medium. The data showed that there were no significant difference among the examined groups (Control: 93.4 ± 1.6%, n = 124; Vitrified + Gly: 91.6 ± 1.2%, n = 125; IVM + Gly: 93.6 ± 1.6%, n = 135; and Vitrified + IVM + Gly: 97.6 ± 1.0%, n = 133) (*P* > 0.05, N = 5) (Fig. 1b). However, adding 1 mM glycine into either vitrification medium or maturation medium significantly increased the percentages of 2-cell embryo (51.4 ± 1.4%, n = 117; 45.7 ± 2.9%, n = 124, respectively) and blastocyst (22.7 ± 1.8%, n = 60; 19.0 ± 1.8%, n = 56, respectively), compared to those in the control group without glycine supplementation (2-cell: 32.8 ± 1.5%, n = 116; blastocyst: 8.7 ± 3.6%, n = 38) (*P* < 0.05, N = 5), but no significant difference was found between glycine supplementation in vitrification medium and that in maturation medium alone (*P* > 0.05, N = 5). Interestingly, adding glycine into both vitrification and maturation medium further enhanced the percentages of 2-cell embryo (65.7 ± 2.2%, n = 130) and blastocyst (32.7 ± 1.7%, n = 85), compared to those of glycine supplementation in either vitrification medium or maturation medium (*P* < 0.05, N = 5). (Fig. 1c,d). It is worth noting that there was no glycine supplementation in the embryo culture medium during all the experiments.

### Loading oocytes with glycine during vitrification and/or maturation improved MII-spindle assembly

**Figure 2:**
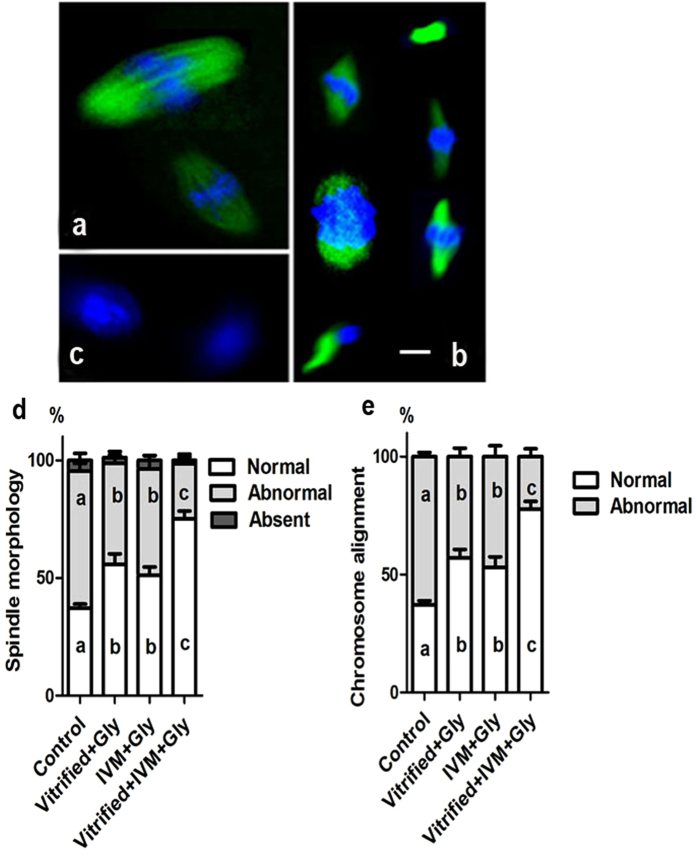
Effects of Glycine addition during vitrification and/or *in vitro* maturation of GV oocytes on the meiotic spindle assembly and chromosomal alignment. (**a, b and c**) Representative fluorescence image of oocyte with either normal meiotic spindle organization and chromosomal alignment (**a**) or abnormal spindle assembly and chromosome alignment (**b**) or without spindle assembly (**c**). (**d**) Comparison of spindle morphology in MII oocyte from different glycine treatment groups. (**e**) Comparison of chromosome alignment in MII oocyte from various glycine treatment groups. Green: spindle, Blue: DNA. Control: without glycine treatment; IVM + Gly: glycine treatment during *in vitro* maturation only; Vitrified + Gly: glycine treatment during vitrification/thawing only; Vitrified + IVM  + Gly: glycine treatment during both vitrification/thawing and maturation *in vitro*. Different low case letters above columns indicate statistical differences at *P* < 0.05. Scale bar (**a–c**) 10 μm.

Correct bipolar spindle assemble is essential for the alignment of all chromosomes at the spindle equator and accurate chromosome segregation in mammalian oocyte, which is served as an important marker of oocyte quality. We speculated that addition of glycine during vitrification and/or maturation increased the formation of blastocyst following COCs freezing, maturation and fertilization *in vitro* as it improved the MII-spindle assembly and chromosome alignment. As shown in Fig. 2. The oocyte spindles were classified into three groups according to morphology: (1) normal spindles with dense, bipolar (barrel-shaped or elliptical) microtubules; (2) abnormal spindles, partial or total disorganization, clumped or dispersed distribution, and multiple spindle-like structures; and (3) absent or no meiotic spindle around the chromosomes. Chromosome alignment was judged into two groups: (1) normal, chromosomes were compact in one area along the equator of the spindle; and (2) abnormal, chromosomes were distributed scatteredly and far away from the equator of spindle (Fig. 2). In parallel with the data that glycine supplementation improved the preimplantation development following COCs vitrification, adding 1 mM glycine into either vitrification medium or maturation medium significantly increased the rates of normal spindle assembly (55.8 ± 4.5%, n = 68; 51.1 ± 3.6%, n = 59, respectively) and chromosome alignment (57.1 ± 3.5%, n = 68; 53.0 ± 4.5%, n = 59, respectively), compared to those in the control group without glycine supplementation (Spindle: 37.2 ± 1.7%, n = 70; Chromosome: 37.2 ± 1.7%, n = 70) (*P* < 0.05, n = 4). No significantly difference was found between glycine supplementation in vitrification medium and that in maturation medium alone. Interestingly, adding glycine into both vitrification and maturation medium further enhanced the percentages of normal spindle assembly (75.0 ± 3.4%, n = 66) and chromosome alignment (77.7 ± 3.3%, n = 66), compared to those of glycine supplementation in either vitrification medium or maturation medium (*P* < 0.05, N = 4) (Fig. 2d,e).

### Glycine addition either in the vitrification/thawing medium or in the maturation medium decreased aneuploidy in the MII oocytes following COCs vitrification

**Figure 3:**
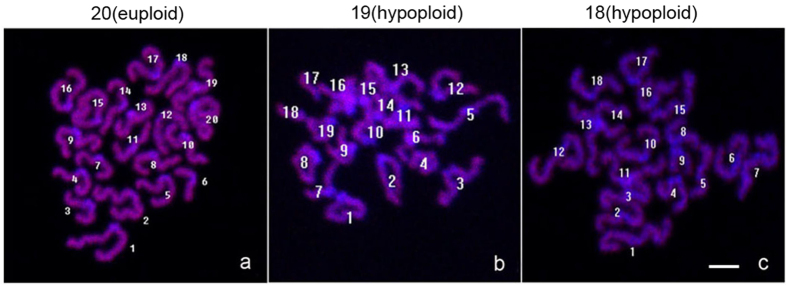
Representative fluorescence images of chromosomal spreads of MII oocytes from the vitrified GV oocytes. (**a**) euploidy; (**b and c**): aneuploidy. Scale bar (**a–c**) 10 μm.

Since addition of glycine enhanced the percentage of normal spindle assembly and chromosome alignment, we further examined whether glycine supplementation could decrease the rate of aneuploidy of MII oocyte following COCs vitrification at GV stage. As expected, the aneuploidy rate was significantly decreased when adding glycine into the vitrification medium and/or IVM medium, compared to the control without glycine addition (*P* < 0.05, N = 5) (Fig. 3 and Table 1). However, no significantly difference was found among addition of glycine into either vitrification medium or maturation medium, or both vitrification and maturation medium (*P* > 0.05, N = 5).

### Glycine supplementation during vitrification of COCs and their subsequent maturation decreased the mitochondrial damage caused by freezing

**Figure 4:**
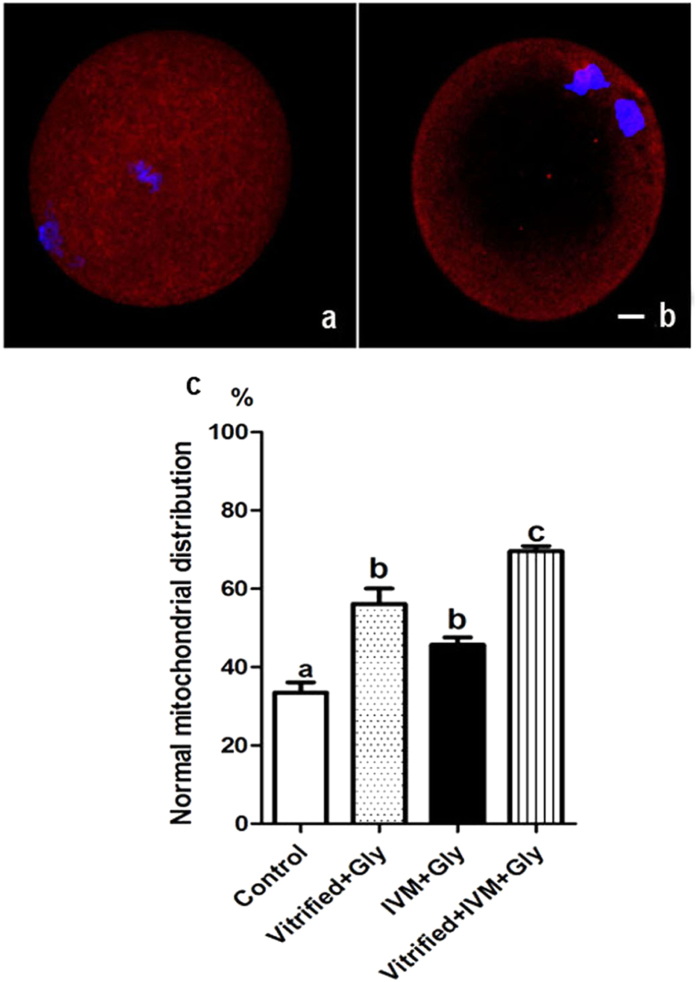
Effects of loading GV oocytes with glycine during vitrification and/or *in vitro* maturation on mitochondrial distribution in the subsequent MII oocytes. (**a**) Example of normal mitochondrial distribution in MII oocyte: mitochondria were distributed throughout the whole cytoplasm including central region. (**b**) Example of abnormal mitochondrial distribution in MII oocyte: mitochondria were mainly localized around cortical area in the oocyte with no or little mitochondrial presented in the center of oocyte. Blue: DNA, red: mitochondria. (**c**) Comparison of mitochondrial distribution in MII oocyte from vitrified COCs with different glycine treatments. Control: without glycine treatment; IVM + Gly: glycine treatment during *in vitro* maturation only; Vitrified + Gly: glycine treatment during vitrification/thawing only; Vitrified + IVM + Gly: glycine treatment during both vitrification/thawing and maturation *in vitro*. Different low case letters above columns indicate statistical differences at *P* < 0.05. Scale bar (**a,b**) 10 μm.

Vitrification usually disrupts the localization and function of mitochondrial in oocytes, which is often blamed for abnormal spindle assembly. We next tested the distribution and function of alive mitochondrial in the MII oocytes following COCs vitrification and their maturation with or without glycine treatment. Roughly, mitochondrial distribution was classified into two categories: (1) mitochondrial diffusing evenly throughout the oocyte, namely evenly distributed mitochondrial, which is found in the majority of non-vitrified MII oocytes^30^ (normal distribution); the (2) mitochondrial mainly surrounding the cortical area, termed peripherally concentrated mitochondrial (abnormal distribution). In consistent with embryo development and spindle assembly data, the oocytes that were treated with glycine either during vitrification (56.1 ± 3.9%, n = 80) or during maturation (45.6 ± 1.9%, n = 79) had a higher chance to be with normal mitochondrial distribution compared to the counterparts that were never treated with glycine (33.4 ± 2.7%, n = 82) (*P* < 0.05, N = 4) (Fig. 4). While little change was seen in the oocytes that were treated with glycine during maturation in improving mitochondrial localization compared to these treated with glycine during vitrification (*P* > 0.05, N = 4). On the other hand, glycine-treatment during both vitrification and maturation could further increase the proportion of MII oocytes with normal mitochondrial distribution (69.6 ± 1.3%, n = 92), when compared with glycine-treatment during either vitrification or maturation alone (*P* < 0.05, N = 4).

### Glycine addition during both vitrification of COCs and their subsequent maturation increased ATP levels in MII oocyte

**Figure 5:**
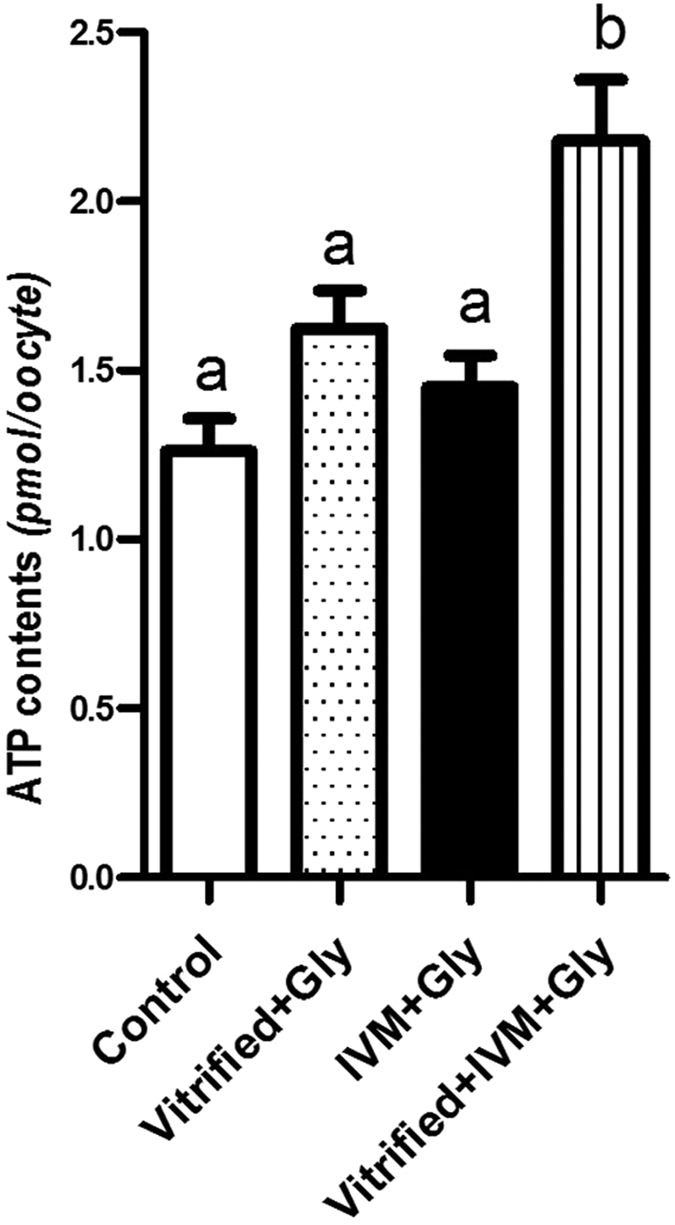
Effects of loading GV oocytes with glycine during vitrification and/or *in vitro* maturation on the ATP levels of the resultant MII oocytes. Control: without glycine treatment; IVM + Gly: glycine treatment during *in vitro* maturation only; Vitrified + Gly: glycine treatment during vitrification/thawing only; Vitrified + IVM + Gly: glycine treatment during both vitrification/thawing and maturation *in vitro*. Data are indicated as mean ± SEM. Different letters above columns indicate significant difference at a level of *P* < 0.05.

The most prominent roles of mitochondrial are to produce the ATP and to regulate cellular metabolism. To further validate whether glycine supplementation during vitrification or maturation lessen the mitochondrial impairment induced by vitrification at GV stage, we evaluated the ATP contents in the resultant MII oocytes. As shown in Fig. 5, glycine addition in either vitrification/warming (1.62 ± 0.1 *p*mol/oocyte) or maturation medium (1.45 ± 0.1 *p*mol/oocyte) did not help to enhance the ATP levels in the resultant MII oocytes compared to the control without glycine addition (1.26 ± 0.1 *p*mol/oocyte). However, the ATP contents in MII oocytes were significantly increased when glycine was added through vitrification/warming and IVM (2.2 ± 0.2 *p*mol/oocyte) compared to that of the counterparts that were never treated with glycine, or co-cultured with glycine either during vitrification/warming or during maturation alone (*P* < 0.05, N = 5).

### Glycine treatment during vitrification and thawing, but not during maturation, inhibited apoptosis in the vitrified GV oocytes and following MII oocytes

**Figure 6:**
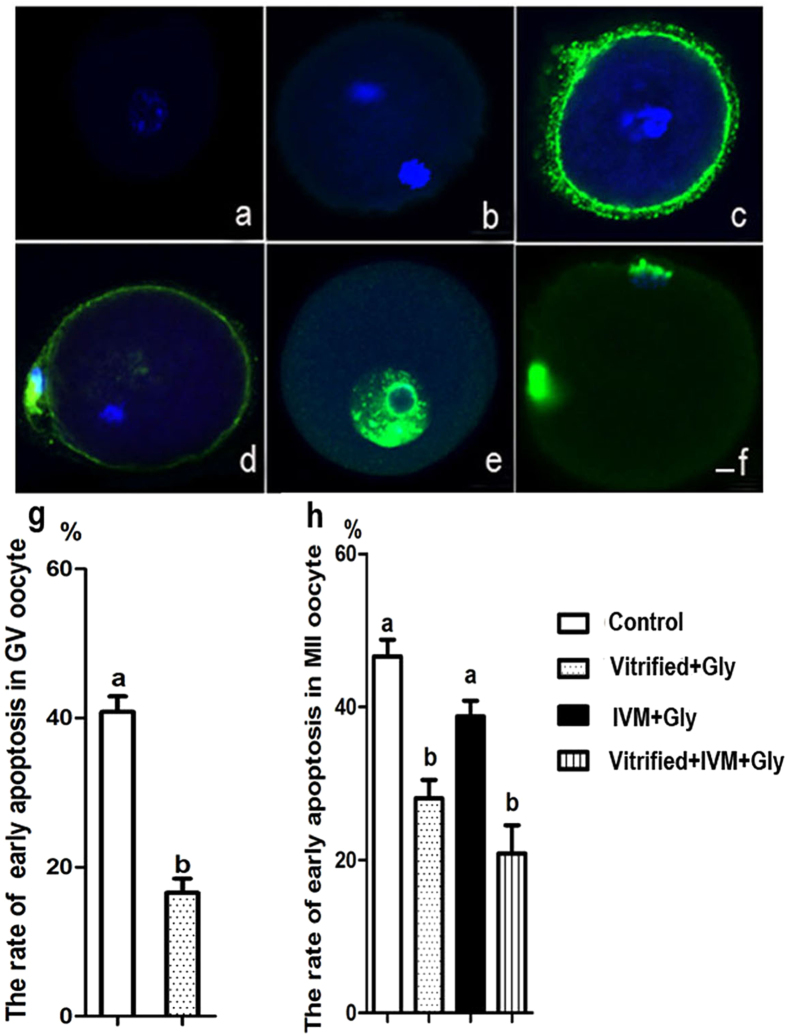
Glycine treatment during GV oocyte vitrification and/or *in vitro* maturation protected against apoptosis in oocyte at both GV and MII stages. (**a–f**) Representative images of early and late apoptosis labeled with Annexin-V (**c**: GV oocyte; **d**: MII oocyte) and TUNEL (**e**: GV oocyte; **f**: MII oocyte) staining, respectively, and their negative control (**a**: GV oocyte; **b**: MII oocyte). Blue: DNA; green: Annexin-V or TUNEL staining. Scale bar (**a–f**), 10 μm. (**g**) The rate of early apoptosis in GV oocyte following vitrirication/thawing with or without glycine treatment. (**h**) The percentage of early apoptosis in MII oocytes following vitrirication/thawing and maturation of COCs with different glycine treatments. Control: without glycine treatment; IVM + Gly: glycine treatment during *in vitro* maturation only; Vitrified + Gly: glycine treatment during vitrification/thawing only; Vitrified + IVM + Gly: glycine treatment during both vitrification/thawing and maturation *in vitro*. Different low case letters above columns indicate statistical differences at *P* < 0.05.

To assess the effect of glycine-treatment during vitrification and/or maturation following COCs freezing on oocyte apoptosis, we stained the resultant oocytes with Annexin-V (early apoptosis) and TUNEL (late apoptosis) staining (Fig. 6). The results exhibited that glycine supplementation in the vitrification and thawing medium (16.6 ± 1.9%, n = 113) decreased the occurrence of early apoptosis in vitrified GV oocytes, compared to the control without glycine supplementation (40.8 ± 2.1%, n = 109) (P < 0.05, N = 4) (Fig. 6c,g). While the apoptotic percentage of MII oocytes from COCs treated with glycine during vitrification/thawing (Vitrified + Gly: 28.1 ± 2.4%, n = 104; Vitrified + IVM + Gly: 20.8 ± 3.7%, n = 106) was dramatically lower than that from COCs without glycine-treatment during vitrification/thawing (Control: 46.6 ± 2.2%, n = 101; IVM + Gly: 38.8 ± 2.1%) (P < 0.05, N = 4) (Fig. 6d,h). Glycine treatment during maturation was less efficient than glycine treatment during vitrification/thawing in preventing the early apoptosis of MII oocytes since no significant difference was found in either groups between COCs with or without glycine treatment during maturation or groups between COCs treated with glycine during vitrification/thawing and these during both vitrification/thawing and maturation (*P* > 0.05, N = 4). In addition, no late apoptosis staining was observed in all examined samples.

## Discussion

Our current results demonstrate for the first time that glycine supplementation during either vitrification/warming or maturation improves cytoplasmic maturation of MII oocytes following vitrified mouse oocytes at GV stage and their subsequent embryo developmental competency. Moreover, addition of glycine during both vitrification/warming and maturation further maximize the beneficial effects of glycine in enhancing the maturation and the resultant embryo development following COCs vitrification. Thus we think that the osmolyte glycine play a protective role during both cryopreservation and subsequent maturation and could be applied as a new nontoxic cryoprotectant for mouse GV oocytes.

There is mounting evidence to suggest that vitrification gives better overall outcomes than slow cooling in mammalian oocytes^31^,^32^,^33^. Compared to controlled rate cooling cryopreservation, vitrification eliminates mechanical injury from ice, transcends the need to find an optimal cooling and warming rate, avoids the need for specialized equipment to control cooling rate, and enables cooling to be rapid enough to outrun chilling injury, but it complicates the osmotic effects of adding and removing high concentrations of cryoprotective agents (CPAs)^34^,^35^. An oocyte initially shrinks rapidly in response to the high extracellular osmolarity, which promotes exchange of intracellular water with permeable CPAs during the vitrification process. As a result, there is an extreme and very rapid increase in inorganic ions in oocytes. Then during the thawing process, exposure to impermeable CPAs, which are critical for success of thawing to prevent cell volume increase above the desired level to death, is reserved until the final step to maintain as large a cell volume as possible. Early studies of osmotic stress focused primarily on minimum or maximum cell volume, particularly because over the minimum or maximum cell volume would lead to cell death. Currently, the issues of cryosurvival have largely been overcome and people are focusing efforts on reducing sublethal damage induced by osmotic stress to increase developmental competence in oocytes or embryos since cell volume homeostasis is a key factor in successful early embryo development. On the other hand, glycine serving as a novel organic osmolyte, but not any of the four known somatic cell organic osmolyte, to overcome the developmental block induced by hypertonic environment was well documented^36^,^37^. Furthermore, the initiation of glycine transport via the GLYT1 transporter that normally occurs when ovulation is triggered *in vivo* or when oocytes are removed from the follicle and placed into *in vitro* culture also initiates the control of cell volume using glycine in fully grown GV oocytes^28^. Thus, as our speculation, glycine supplementation during vitrification and maturation exerts its advantage roles in reducing the osmotic stress when adding and removing high concentrations of cryoprotective agents during vitrification and thawing.

The osmotic stress during dehydration and rehydration of vitrification process is a well-documented detrimental factor in oocytes at the GV stage, which has been reported in mice^14^, porcine^38^ and human oocytes^39^, resulting in the irreversible damage to the organelle and ultrastructure, including abnormal spindle assembly and chromosome segregation, mis-distributed mitochondrial during oocyte meiosis, and continued development^40^,^41^. In consistent with our study, addition of 1 mM glycine, a class of diverse, small, neutral organic compounds accumulated by cells to provide intracellular osmotic support to replace ions that can disrupt cellular physiology at higher concentrations^42^,^43^, to the vitrification solutions improved the ability of the mouse MII oocytes to maintain their mitochondrial physiology and increase the blastocyst development^29^. It has been suggested that meiotic spindle assembly, which is critical for correct sister chromatids segregation and subsequent embryo development^10^,^44^, have been associated with mitochondrial distribution and activity in both human and mouse oocytes^45^. In our study, as expected, glycine supplementation during vitrification and maturation following mouse COCs vitrification enhanced the meiotic spindle assembly, chromosome alignment, ATP contents, and reduced the percentage of aneuploidy in the resultant MII oocytes.

Moreover, studies have reported that various cell types, including cardiac fibroblasts^46^, medullary epithelial cells^47^ and rat alveolar type II cells^48^ were apoptotic when cultured in the hyperosmotic medium. Although no direct link has been established between osmotic stress and oocyte apoptosis, cell death through apoptosis were also happened after oocyte vitrification^49^,^50^,^51^. Glycine prevents the cells against apoptosis has been reported in rat endothelial cells^52^, mice neuronal cells^53^ and chick embryos^54^. For mouse oocytes, we demonstrated for the first time that the glycine supplementation during vitrification/thawing could inhibit early apoptosis (detected by Annexin V binding) in vitrified GV oocytes. However, whether glycine addition during embryos culture could further enhance the outcome of mouse COCs vitrification has yet to be established.

Oocyte cryopreservation is considered relatively inefficient since, at least in part, the oocyte is a large single cell with relatively low surface-to-volume ratio. However, it has numerous advantages over embryo cryopreservation. Our current study suggests that optimization of cell volume regulation by adding organic osmolyte of glycine during freezing-thawing and/or maturation *in vitro* could improve other mammalian and human oocyte vitrification at GV stage.

## Materials and Methods

Unless otherwise specified, all chemicals used in the study were purchased from Sigma Chemical Co. (St. Louis, MO, USA).

### Mouse

All animal experiments were conducted exactly in accordance with the guide to the Care and Use of Experimental Animal issued by the Animal Ethics Committee of Institute of Special Animal and Plant Sciences, Chinese Academy of Agricultural Sciences (Permit Number: 2014–0035). In addition, these experimental procedures had been approved by the committee before we did our experiments. Kunming white mouse, a native breed widely used in biological research in China, was housed in a temperature-controlled room with 14 h light/10 h dark cycle, fed with commercial diet and water ad libitum. Mice of 5–6 weeks old were injected intraperitoneally each with 5 IU equine chorionic gonadotropin (eCG), and sacrificed 45–47 h later by cervical dislocation for oocyte collection.

### Collection of oocytes

Fully-grown GV-stage oocytes surrounded by cumulus cells (CCs), named COCs, were isolated by puncturing large antral follicles with a pair of 26-gauge needles. Only COCs with more than three layers of CCs and homogenous cytoplasm oocytes ~80 μm in diameter were selected for the following experiments.

### Vitrification and thawing of oocytes

COCs were vitrified by cryoloop (Hampton Research company, USA) method as previously described^55^. Briefly, the base medium (BM) of vitrification and warming solutions was MEM-alpha supplemented with 10% FBS. For vitrification, COCs were firstly transferred into equilibrium solution (BM containing 7.5% ethylene glycol (EG), 7.5% dimethylsulfoxide (DMSO), and 0.25 M trehalose) for 3 min. Then COCs were moved into a vitrification solution (BM containing 15% EG and 15% DMSO). Three to five COCs were placed with as little solution as possible on the thin film, which was formed by surface tension following the cryoloop was dipped into the vitrification solution, and directly plunged into liquid nitrogen. The best vitrification results could be got if the whole process was less than 1 min from cells were transferred into vitrification medium to they were vitrified. To warm the vitrified COCs, the cryoloop containing vitrified COCs was immediately dipped into warming solution I (BM supplemented with 1 M trehalose) for 3 min. Then COCs were transferred into warming solution II (BM supplemented with 0.5 M trehalose) and BM, respectively, for 3 min each. The whole thawing procedures were done at 37 °C. The viability of COCs was determined morphologically by whether they had evenly granulated cytoplasm with apparent perivitelline space (viable) or degenerated cytoplasm without perivitelline space (nonviable). Only viable COCs were used for the following experiments.

### Oocytes maturation *in vitro*

The maturation medium was TCM-199 medium (Gibco, Grand Island, NY, USA) supplemented with 10% (v/v) fetal calf serum (FCS) (Gibco, Grand Island, NY), 1 mg/ml 17 β-estradiol, 24.2 mg/l sodium pyruvate, 0.05 IU/ml FSH, 0.05 IU/ml LH, 10 ng/ml EGF. A group of 20–30 COCs were cultured in a 100 μl drops of maturation medium at 37 °C in an atmospehere of 5% CO_2_ and saturated humidity for 16 h.

### Glycine supplementation

Glycine was dissolved in the medium at a final concentration of 1 mM and sterilized by filtration.

### Fertilization and embryo culture

Sperms were collected from the cauda epididymis of fertile male mice (Kunming white mice) and were placed at the bottom of a test tube containing T6 medium supplemented with 10 mg/ml bovine serum albumin (BSA). After 3–5 min, these highly motile spermatozoa in the supernatant medium were transferred and capacitated in the same medium for 1.5 h. After being washed with fertilization medium (T6 containing 20 mg/ml BSA), groups of 20–30 COCs were transferred to 100 μl droplets of pre-warmed fertilization medium and fertilized by 1 × 10^6^/ml capacitated sperms for 6 h. After fertilization, these supposed fertilized eggs were firstly cultured in the CZB medium until 4-cell embryo stage and then moved to CZB medium supplemented with 5.5 mM glucose for further development to blastocyst stage. All culture conditions were at 37 °C under 5% CO_2_ in humidified atmosphere.

### Meiotic spindle assembly and chromosomal alignment

Before immunofluorescence staining of spindle, oocytes were treated with 300 μg/ml hyaluronidase for 5 min at 37 °C and denuded of cumulus cells by repeated pipetting in an M2 medium. Denuded oocytes were fixed in a solution containing 2% paraformaldehyde and microtubule stabilizing buffer^56^ for 60 min at room temperature. Then, oocytes were transferred into blocking buffer (PBS supplemented with 1% BSA and 0.01% Triton X-100) for 1 h at room temperature and incubated with mouse anti-alpha-tubulin-FITC monoclonal antibody (Sigma) diluted 1:200 in the blocking buffer for 60 min at room temperature. After washing thoroughly, the oocytes were transferred to a small drop of Prolong Antifade mounting medium containing 4, 6-diamidino-2-phenylindole (DAPI) and mounted on microscope slides.

### Mitochondrial distribution

COCs were removed cumulus cell and then were cultured in M2 medium containing 200 μM Mitotracker Red (Mitotracker Red FM; Molecular Probes, Eugene, OR, USA) for 30 min at 37 °C. After washing, oocytes were fixed in 4% paraformaldehyde in PBS for 20 min. After permeated by 0.5% Triton X-100 for 20 min, oocytes were transferred into a drop of Prolong Antifade mounting medium containing DAPI on a microscope slide and covered with a coverslip.

### Oocyte chromosome spreads

Chromosome spreads were carried out according to Hodges and Hunt^57^ with minor modifications. In brief, the zona pellucida of oocytes was removed by treatment with 0.5 units/μl pronase at 37 °C for 20 sec. Zona-free oocytes were rinsed in M2 medium to detach polar bodies. The oocytes were individually plated onto Plus-charged histology slides that were covered by the fixative solution (PBS containing 4% paraformaldehyde (w/v), 0.15% triton X-100 (v/v) and 3 mM dithiothreitol, pH 9.2). After these slides were air-dried, they were incubated with 10 μg/ml PI and DAPI for 10 min. Finally, these slides were mounted in the Prolong Antifade mounting medium following washing thoroughly with PBS. Chromosomes were observed under a confocal microscope.

### Measurement of ATP Contents in MII oocytes

After 16 h of IVM, oocytes were treated with 300 μg/ml hyaluronidase for 5 min at 37 °C and denuded of cumulus cells by repeated pipetting in an M2 medium. Only matured oocytes with first polar body were selected for ATP assay. 30 oocytes in each group were snap-frozen in a microfuge tube containing 200 μl of ultrapure water and stored at −80 °C. ATP contents in oocytes were determined by using the assay kit (Bioluminescent Somatic Cell Assay Kit, FL-ASC, St Louis, MO) based on the luciferin–luciferase reaction as previously described^56^. The bioluminescence of each sample was measured by high-sensitivity luminometer (Berthold LB 9508, Germany) including a standard curve with different ATP concentrations in each assay.

### Annexin V staining

Early-stage apoptosis of oocytes was detected using Annexin V FLUOS Staining Kit (Roche, Germany). Briefly, denuded oocytes were transferred into liquid mixture containing Annexin-V buffer, Annexin-V fluos, and PI for 15 min at 37 °C in the dark. After three washes in PBS containing 0.1% Tween 20 and 0.01% Triton X-100, oocytes were mounted with the Prolong Antifade mounting medium containing DAPI on a microscope slide and covered by a coverslip. PI used in this experiment was to distinguish live cells from dead cells. PI can only pass through the cell when cytoplasmic membrane has lost its integrity. The oocytes were divided into three groups according to Anguita *et al*.^58^ (1) viable oocytes: non Annexin-V staining; (2) Early apoptotic oocytes: oocytes membrane were stained by Annexin-V; and (3) necrotic oocytes: PI positive red nuclei.

### TUNEL staining

DNA fragmentation was determined by TUNEL staining (Roche, Germany). Denuded Oocytes were fixed with 4% paraformaldehyde in PBS for 1 h at room temperature. Then oocytes were permeabilized in PBS containing 0.1% Triton X-100 and 0.1% sodium citrate for 30 min at room temperature, followed incubation for 1 h at 37 °C in the dark with TUNEL reaction mixture (containing fluorescein isothiocyanate conjugated dUTP and terminal deoxynucleotidyl transferase). Finally, these oocytes were mounted in the Prolong Antifade mounting medium following washing thoroughly with PBS containing 0.1% Tween 20 and 0.01% Triton X-100. During each experiment, a group of oocytes treated with RQ1 RNase-free DNase (50 U/ml) at 37 °C for 1 h was used as a positive control; while another group of oocytes incubated with TUNEL reaction mixture without the terminal deoxynucleotidyl transferase was used as a negative control.

### Laser confocal microscope

All immunofluorescence stained oocytes were observed under a Nikon laser scanning confocal microscope (Nikon C2 plus Si). When DAPI fluorescence was monitored, the excitation light wavelength was 405 nm and emission light wavelength was 450–480 nm. When FITC, Annexin V and TUNEL fluorescence was monitored, the excitation light wavelength was 488 nm and emission light wavelength was 515–535 nm. Both PI and Mitotracker Red fluorescence was scanned, the excitation light wavelength was 543 nm and emission light wavelength was 590–630 nm. Images were merged by Nikon Confocal Software.

### Data analysis

Each experiment was repeated at least three times. Statistical analyses were performed using Statistics Package for Social Science (SPSS 17.0; SPSS Inc., Chicago, IL). All data were expressed as mean ± SEM. N indicates the number of independent experiments, while n indicates the total number of individual oocytes or embryos. The percentage data were arc-sine transformed before statistical analysis. Data were analyzed by either student’s t-test (two groups) or one-way ANOVA followed by the Duncan multiple comparison test (four groups). All results were considered to be statistically significant at a level of *P* < 0.05.

## Additional Information

**How to cite this article**: Cao, X.-Y. *et al*. Glycine increases preimplantation development of mouse oocytes following vitrification at the germinal vesicle stage. *Sci. Rep.* **6**, 37262; doi: 10.1038/srep37262 (2016).

**Publisher’s note:** Springer Nature remains neutral with regard to jurisdictional claims in published maps and institutional affiliations.

## Figures and Tables

**Table 1:** Aneuploidy rates in mouse MII oocytes following vitrified COCs with different glycine treatments.

Groups	No. of oocytes examined	No. of oocytes with	
		Haploidy (%)	Aneuploidy (%)
Control	73	66 (90.5 ± 1.3)^a^	7 (9.5 ± 1.3)^a^
IVM + Gly	79	76 (96.3 ± 1.6)^b^	3 (3.7 ± 1.6)^b^
Vitrified + Gly	81	78 (96.4 ± 1.6)^b^	3 (3.6 ± 1.5)^b^
Vitrified + IVM + Gly	82	80 (97.9 ± 1.3)^b^	2 (2.2 ± 1.3)^b^

Note: Different letters within the same column indicate statistical differences at *P* < 0.05 by one-way ANOVA followed by the Duncan multiple comparison test (N = 5). Control: without glycine treatment; IVM + Gly: glycine treatment during *in vitro* maturation only; Vitrified + Gly: glycine treatment during vitrification/thawing only; Vitrified + IVM + Gly: glycine treatment during both vitrification/thawing and maturation *in vitro*.
